# Supplementary material for: Field-Free Spin–Orbit Torque Driven Switching of Perpendicular Magnetic Tunnel Junction through Bending Current
Source: Nano Lett. 2023 Jun 9;23(12):5482–9. doi: 10.1021/acs.nanolett.3c00639 (PMC10311595; doi:10.1021/acs.nanolett.3c00639)
Supplement: Supplementary file 1 — nl3c00639_si_001.pdf [file nl3c00639_si_001.pdf]

# Supporting Information for Field−Free Spin−Orbit Torque Driven Switching of Perpendicular Magnetic Tunnel Junction through Bending Current

*Vaishnavi Kateel*[†, ‡, *], *Viola Krizakova*[§], *Siddharth Rao*[†, *], *Kaiming Cai*[†], *Mohit Gupta*[†], *Maxwel Gama Monteiro*[†, ‡], *Farrukh Yasin*[†], *Bart Sorée*[†, ‡], *Johan De Boeck*[†, ‡], *Sebastien Couet*[†], *Pietro Gambardella*[§], *Gouri Sankar Kar*[†], *Kevin Garello*[†, ||, *]

[†]IMEC Kapeldreef 75, B-3001 Leuven, Belgium,

[‡]Department of Electrical Engineering ESAT, KU Leuven, Kasteelpark Arenberg 10, B-3001 Leuven

[§]Department of Materials, ETH Zurich, 8093 Zürich, Switzerland

[||]Univ. Grenoble Alpes, CEA, CNRS, Grenoble INP, SPINTEC, 38000 Grenoble, France

I. Magnetic properties of bending structure

II. Finite element method simulation

III. SOT induced switching in bending structures

  i. Experimental setup for DC SOT switching

  ii. Experimental setup for pulsed SOT switching

  iii. Field-free switching

IV. Micromagnetic Simulation:

  i. Switching mechanism of bending structures

  ii. Switching robustness of bending structures

  iii. Change in simulation parameters.

V. Influence of SAF Hard layer

VI. Bending structure design analysis

# I. Magnetic properties of bending structure

The devices show a square hysteresis loop with strong PMA for all the MTJ sizes. Statistics of magnetic properties of the 20 measured MTJs are listed in SI Table 1. The perpendicular anisotropic field $B_k$ and thermal stability factor $\Delta$ are calculated by sweeping the external magnetic field along the $z$-direction. Then, the switching probability is fitted to equation 1, where $r$ is the magnetic field sweep rate and $f_0$ is the attempt frequency (in our case~1 $ns^{-1}$)[1].

$$P_{SW}(B_z) = \frac{-B_k f_0 \sqrt{\pi}}{2r\sqrt{\Delta}} \operatorname{erfc}\left[\sqrt{\Delta}\left(1 - \frac{B_z}{B_k}\right)\right] \qquad (1)$$

| MTJ size (nm) | RA ($\Omega\mu m^2$) | $B_c$ (mT) | $B_{off}$ (mT) | $B_k$ (mT) | $\Delta$ (kT) | $R_{SOT}$ ($\Omega$) |
|---|---|---|---|---|---|---|
| 60 | 60 ± 6 | 61.6 ± 7.8 | -6.8 ± 4 | 180 ± 3.4 | 49 ± 15 | 4350 ± 140 |
| 80 | 51 ± 3 | 80 ± 7.4 | -7 ± 3.1 | 217 ± 17 | 52 ± 6 | 3175 ± 66 |
| 100 | 40 ± 2 | 89 ± 7.4 | -6.8 ± 2.8 | 248 ± 14 | 56 ± 4 | 3160 ± 100 |

*SI Table 1*. Static magnetic properties of bending structure with varying MTJ sizes

# II. Finite element method simulation

The present design of the bending structures consists of the SOT channel with two bents, and in one of the bends, the MTJ is placed closer to the outer corner, as shown in the SI Figure 1a. The asymmetric charge current and temperature profile due to Joule heating are simulated with COMSOL Multiphysics software AC/DC module using the current continuity model and Ohms law for the SOT-MRAM device (SI Figure 1b). A SOT-MRAM 3D model is created for a 60 nm MTJ size with stack composition as listed above (all the dimensions like the device) with the top copper electrode, SOT hard mask, MTJ hard mask, and VIA contacts to mimic the actual device. The model is insulated at all surfaces except the top electrode and the two VIA contacts with $SiO_2$. The MTJ location on the SOT channel is taken from the top view of TEM. A

tetrahedral mesh size varying from 5.34 nm to 73.4 nm is used for the model with dimensions of 0.3×1.5×0.5 $\mu m^3$. The simulation is performed by applying $V_{SOT} = 2.5$ V (Similar to the experimental requirement for DC switching) to one of the VIA, and the other VIA is grounded; the top electrode is compensated with a bias such that there is no drop across MTJ ($V_{\mathrm{Comp}} = 0.304$ V). The curvature in the SOT channel causes an inhomogeneous current density, with the inner corners of the MTJ having a higher current density flowing than the outer corner (SI Figure 1c).

The charge current at the bend helps create a gradient in the spin current, and the bending of the current also causes a spatially varying spin polarization. Thus, it generates a spatially varying torque in the $xy$ plane, causing symmetry breaking. As the spin current is generated in the W SOT channel due to the spin Hall effect, it can be given as $\boldsymbol{J_S} = \theta_{SH}\boldsymbol{J_C}$, in our case, the effective spin Hall angle is −0.43[2]. The higher spin current supports creating a fixed nucleation point in the FL closest to the inner corner of the SOT channel (SI Figure 1d). The inhomogeneous spin polarization ($\sigma \sim \hat{z} \times J_c$) is created due to the bending of the SOT channel. The large inhomogeneous current flowing in the SOT channel contributes to Joule heating, causing a temperature gradient of 40 K across the MTJ. We also performed COMSOL simulation for an optimized design with shorter L-shaped SOT channel (SI Figure 1e). We observed no change in trends of the current density gradient or temperature gradient below the MTJ. The extra arm used in our present design only contributes to increasing the resistance of the channel and does not play any role in the FFS of bending structures. Thus, we used the optimized design as input for our micromagnetic and DTCO modeling.

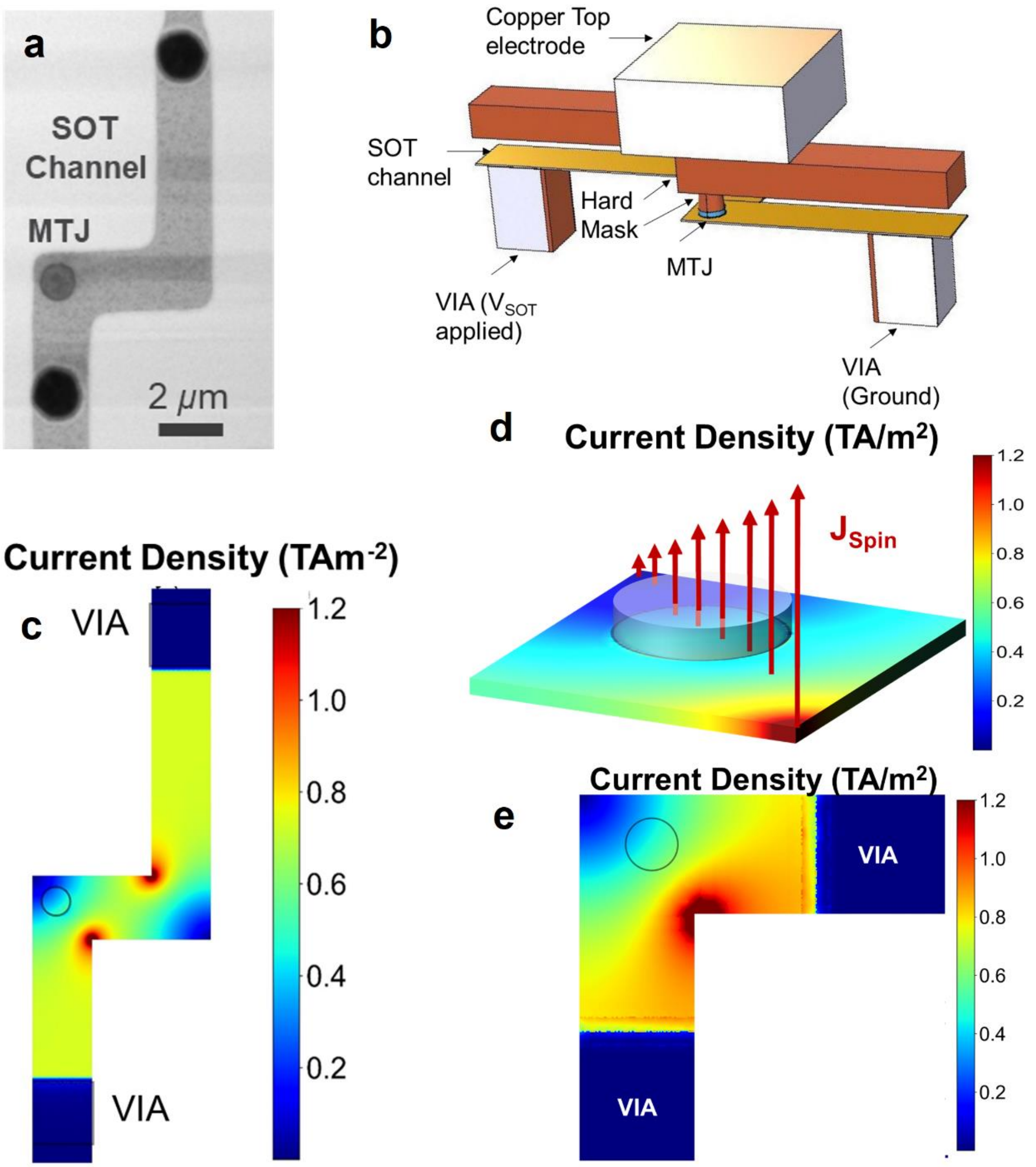


*SI Figure 1. COMSOL Simulation for SOT MRAM. (a)* Top view TEM for the SOT channel showing various components. *(b)* SOT-MRAM model with all simulated features of the actual SOT device and surrounded by $SiO_2$ (not shown) for creating isolation. *(c)* Inhomogeneous charge current density in the SOT channel. *(d)* Schematic showing the possibility of a fixed nucleation site with a higher spin current at the inner corner due to the $J_c$ variation. *(e)* Current density for the optimized L-shaped bending structure.

## III. SOT induced switching in bending structures.

### i. Experimental setup for DC SOT switching.

A direct current is injected through the SOT channel, and the resistance of MTJ is read by applying a current of 5 μA. During the current injection, the MTJ is kept at high resistance such that there is no drop across the MTJ.

An additional in-plane magnetic field along the $x$ and $y$ directions is used for the angular in-plane field-induced SOT switching. The angular variation of the in-plane field is generated by varying the ratio of $\frac{B_X}{B_Y}$ and direction with keeping the $|B| = \sqrt{B_X^2 + B_Y^2} = 25$ mT.

### ii. Experimental setup for pulsed SOT switching.

The electrical set-up for pulsed switching (as reported in ref.[3]) consists of a single pulse generator with two independent output $V_{SOT}$ and $V_{COMP}$ connected to the RF terminal of bias Tee 1 and bias Tee 2, the other input of bias Tee 1 and bias Tee 2 is connected to the individual DC supply for post pulse resistance read. The bias Tee 1 and 2 outputs are connected to the SOT channel and MTJ, respectively, such that there is no delay between the two pulses. According to our sign convention, $V_{COMP} > 0$ is for the current flowing in the $-z$ direction, and $V_{SOT} > 0$ is for the current flowing in the $-y$ and $+x$ directions (SI Figure 2a).

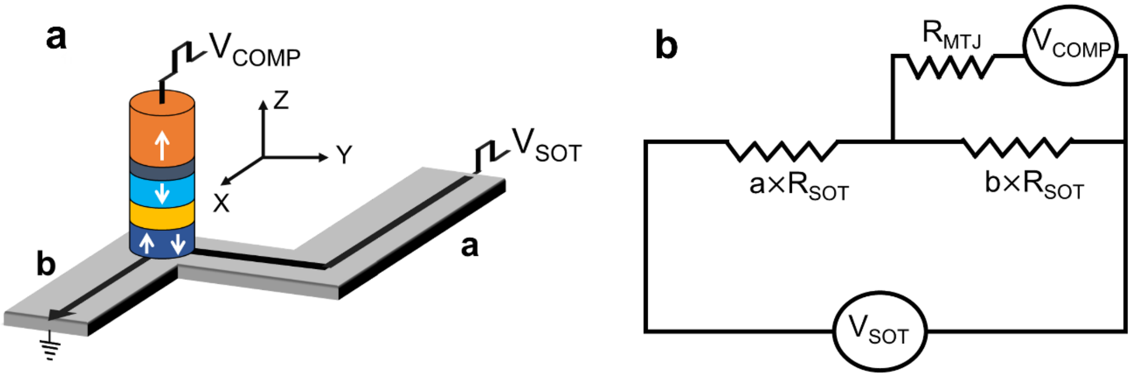


*SI Figure 2. Switching circuit for pulsed current. (a)* Schematic for bending structure devices. *(b)* Equivalent circuit model.

---

Due to the asymmetric position of MTJ on the SOT channel, the resistance equivalence circuit for the bending structure would depend on the dimension of MTJ and the length of the SOT channel (SI Figure 2b). Therefore, the resistance of the SOT channel on the two sides of MTJ is written as the function length of the SOT channel (as $R \propto L$), with $a + b = 1$ where $a$ is the longer arm of the SOT channel. From the circuit analysis, the relation of the $V_{COMP}$ and $V_{SOT}$ is given by.

$$V_{MTJ} = \frac{V_{\mathrm{COMP}} - bV_{\mathrm{SOT}}}{\left(ab \times R_{\mathrm{SOT}}/R_{MTJ} + 1\right)}$$

For a pure SOT-induced switching, the potential drop across the MTJ ($V_{MTJ}$) should be zero; thus, the $V_{\mathrm{COMP}}$ is a function of the length of the SOT channel and given by $bV_{\mathrm{SOT}}$. Considering the impedance mismatch, the $V_{\mathrm{Applied}} = 2 \times \frac{R_{DUT}}{R_{DUT}+50} V_{\mathrm{applied-input}}$. Using this theory, the $V_{\mathrm{COMP}}$ and $V_{\mathrm{SOT}}$ can be written as $V_{\mathrm{COMP}} = 2 \times \frac{R_{MTJ}}{R_{MTJ}+50} V_{\mathrm{COMP-input}}$ and $V_{SOT} = 2 \times \frac{R_{SOT}}{R_{SOT}+50} V_{SOT-input}$, respectively. For the 60 nm device, the measured $R_{\mathrm{MTJ}}$ = 16-32 kΩ and $R_{\mathrm{SOT}}$ = 4700 Ω; thus, a $V_{\mathrm{COMP}} = 0.34\ V_{\mathrm{SOT}}$ is needed for having pure SOT-induced pulsed switching.

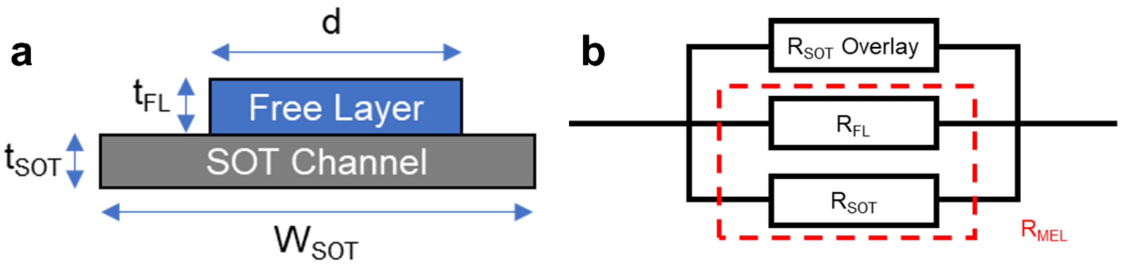


*SI Figure 3. Current density calculation for SOT-MRAM. (a)* Side view of the SOT channel and Free layer. *(b)* Electrical equivalent circuit for SOT-MRAM.

The current density required for switching in the device is calculated by considering the current shunting effect in the free layer and the sides of the SOT channel with no free layer because of the comparable free layer and SOT channel resistivities and wider SOT channel compared to free layer width, respectively (as shown in SI Figure 3). The current density flowing through the memory element $J_{MEL}$ (FL and SOT channel below FL) is calculated using a parallel resistor model (Equation 2).

$$R_{MEL} = \frac{R_{SOT} \times R_{FL}}{R_{SOT} + R_{FL}}$$

$$R_{TOT} = \frac{R_{MEL} \times R_{SOT\ Overlay}}{R_{SOT\ Overlay} + R_{MEL}}$$

$$J_{MEL} = \frac{R_{TOT}}{R_{MEL}} \times \frac{I}{d \times (t_{SOT} + t_{FL})} \quad (2)$$

The dimensions are $d$ = 60 nm, $t_{FL}$ = 0.8 nm, $t_{SOT}$ = 3.5 nm, and $W_{SOT}$ = 190 nm with their resistivity as $\rho_{SOT} = \rho_{\mathrm{SOT\text{-}overlay}}$ = 160 µΩ·cm and $\rho_{\mathrm{FL}}$ = 120 µΩ·cm. All the current density

calculation in this article is done by the abovementioned method. For a 60 nm MTJ device, with $I$ = 1 mA, the calculated $J_{MEL}$ = $1.91\times10^{8}$ A $cm^{-2}$.

iii. Field-free switching

The bending structure with W SOT channel (Figure 1f) and Pt SOT channel with a synthetic ferromagnetically coupled Co/Ru/Co/spacer/CoFeB FL (stack details in ref [4]) switch deterministically with direct current (SI Figure 4a). The zero in-plane field switching loops for both SOT channels show opposite switching polarity due to their oppositive spin Hall angle (Pt and W have positive and negative spin Hall angle, respectively). The directional nature of the switching loop is in accordance with the spin Hall angle of the SOT channel, thus further confirming that the switching in bending structure is a SOT-induced phenomenon and not dominated by the Oersted field (as for both the structures, the current flow direction determines the Oersted field).

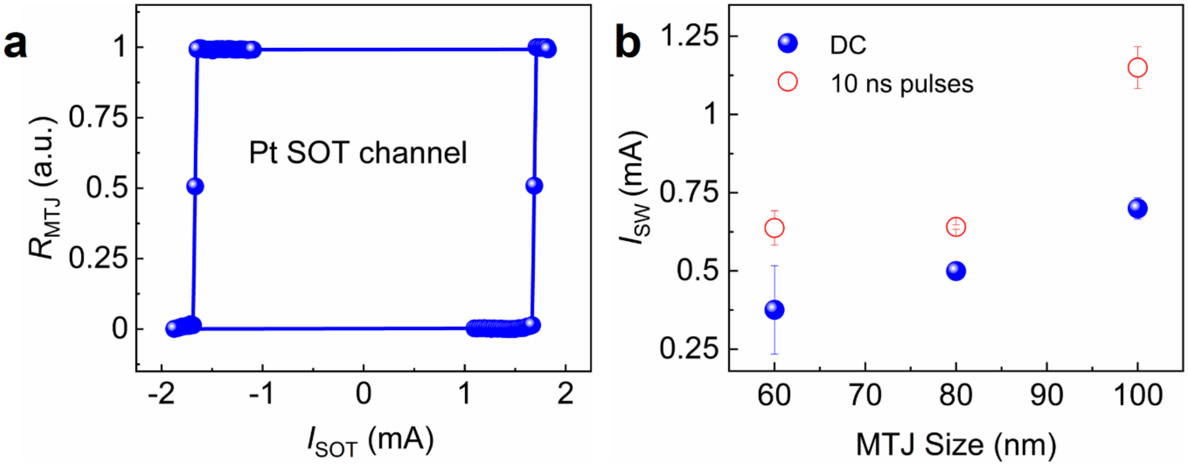


*SI Figure 4. Demonstration of FFS in bending structures. (a)* FFS in Pt SOT channel with coupled FL showing CCW loop. *(b)* Switching current as a function of MTJ size for 10 ns pulse current and DC.

In SOT-MTJ devices, the MTJ is compensated ($V_{COMP}$) such that there is no flow of STT current due to voltage drop across the MTJ. $V_{COMP}$ varies for various MTJ sizes due to inhomogeneous placement of MTJ on the SOT channel, $V_{COMP}$ of 0.34 $V_{SOT}$, 0.32 $V_{SOT}$, and 0.24 $V_{SOT}$ is required for 60 nm, 80 nm, and 100 nm devices, respectively. SOT-induced switching at various MTJ sizes with a 10 ns current pulse increases the switching current with an increase in MTJ size (SI Figure 4b). This increase is due to the bigger magnetic volume of the FL, which causes a stronger anisotropy field in bigger MTJ. This impacts nucleation energy, which is the main contributing factor to the energy consumption of SOT-MRAM. The switching in our devices occurs by reverse domain nucleation followed by domain wall propagation. The bending structures with the W SOT channel cannot switch below 5 ns for 60 nm and 10 ns for 80 nm and

100 nm due to increased bias on the SOT channel, which causes electromigration and SOT channel breakdown. This large bias requirement is not the limitation of bending structure but the present design for our devices. With an optimized design, it should be possible to achieve reliable and faster switching down to sub-nanosecond times in bending structures.

## IV. Micromagnetic Simulation:

The Micromagnetic simulation was performed in Mumax3, a micromagnetic solver based on finite difference method[5], inputting the inhomogeneous 2D map of current density. The device geometry is taken from the top view TEM image shown in SI Figure 1a). We have discretized the geometry with cells of $1\times1\times0.9\ \mathrm{nm}^3$, considering the parameters averaged along the *z*-direction. The $M_s$ = 1.1 MA $m^{-1}$ and $K_u$= 845 kJ $m^{-3}$ at 300 K is calculated similarly to our previous work[2,6]. The SOT effective field ($\theta_{SH} = -0.42$, $B_{FL}/B_{AD} = -0.3$ is calculated from second-harmonic SOT measurement[2]), the DMI strength =0.06 mJ/$m^2$ (stabilizing the right-handed Neel domain wall), exchange interaction strength = 13 pJ/m[6] and Oersted field contributions are considered.

To further understand and verify our hypothesis of deterministic nucleation and symmetry breaking in bending structures, micromagnetic simulations are performed with spatial variation of magnetic properties and location of MTJ, pulse width, and non-standard simulation parameters. These variations give insight into their role in the switching dynamics of bending structures.

### i. Switching mechanism of bending structures

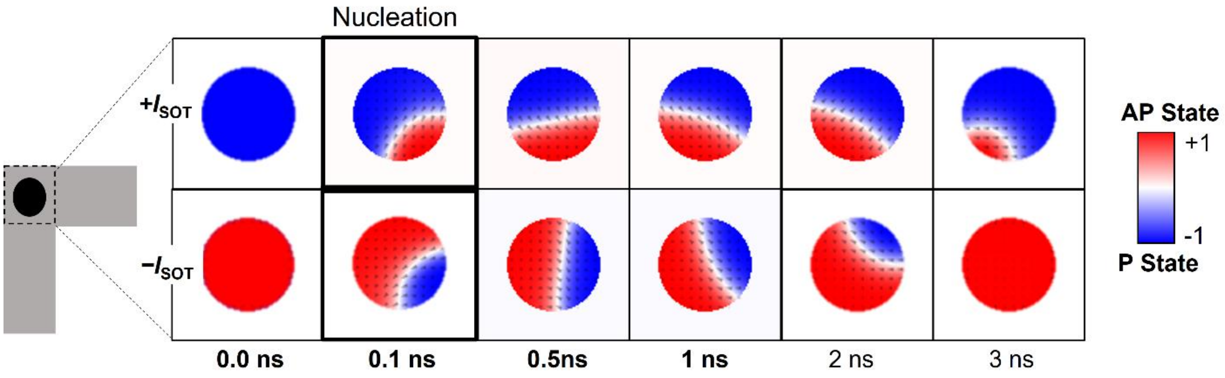


*SI Figure 5. Non-switching case for bending structures:* Magnetization snapshot for $M_z = -z$ (+z) when $I_{SOT} > 0$ (<0).

In Figure 3b,d and SI Figure 5, we see that there is nucleation of reverse domain for the all the combination of initial $M_z$ and $I_{SOT}$ direction indicating the fixed nucleation occurs at inner corner. This is further explained schematically in SI Table 2 with formation of Neel's domain wall at the inner corner. Interestingly, we observe domain wall propagation followed with complete magnetization reversal only for fixed combination of initial $M_z = +z$ $(-z)$ and $+I_{SOT}$ $(-I_{SOT})$. In the opposite combination of initial $M_z$ and $I_{SOT}$ direction the domain wall propagation causes the FL to end up in the initial $M_z$ state. Complete reversal requires the domain wall to maintain its chirality when orienting along the spatially varying spin polarization as shown in SI Table 3. The fixed switching polarity indicates breaking of time reversal symmetry in our system.

s

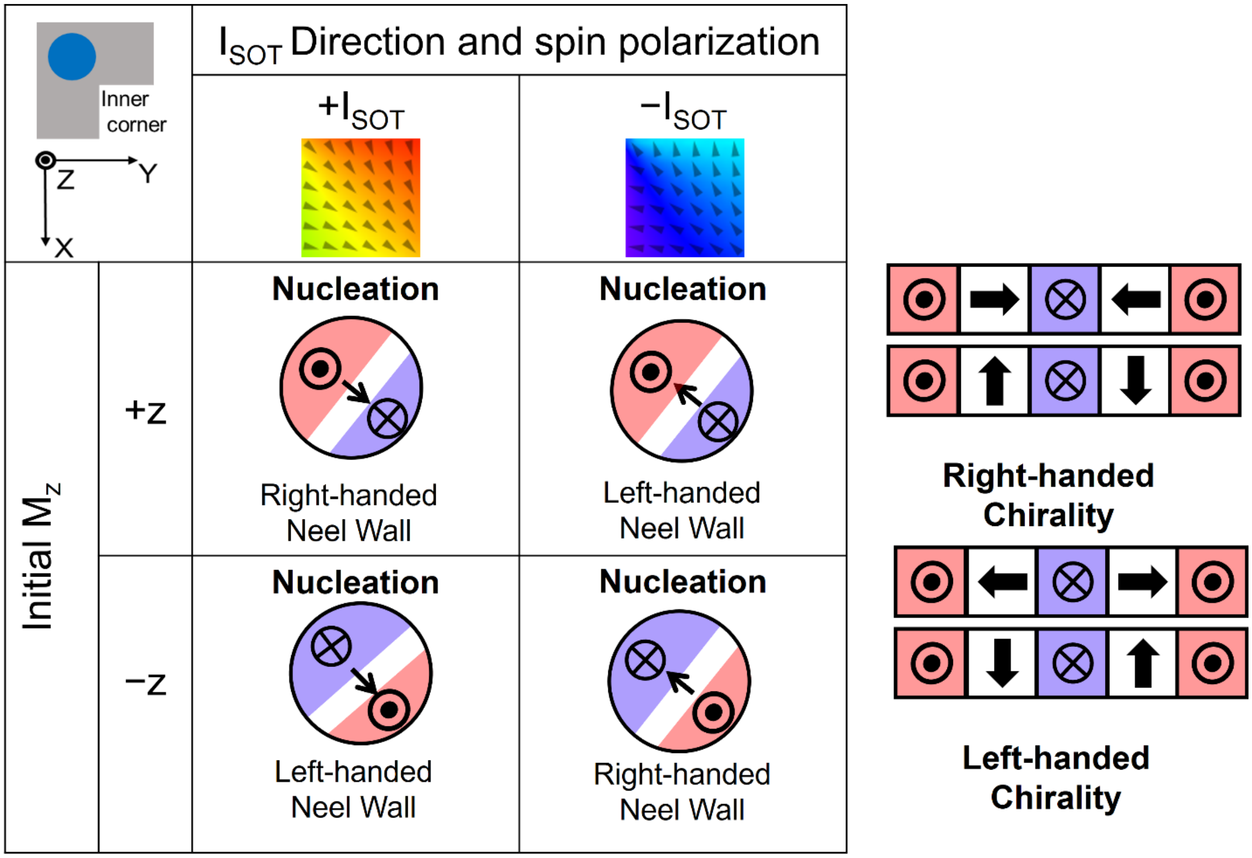


*SI Table 2*. Fixed nucleation at the inner corner for all the combination of $I_{SOT}$ and initial $M_z$.

showing a Neel Domain wall.

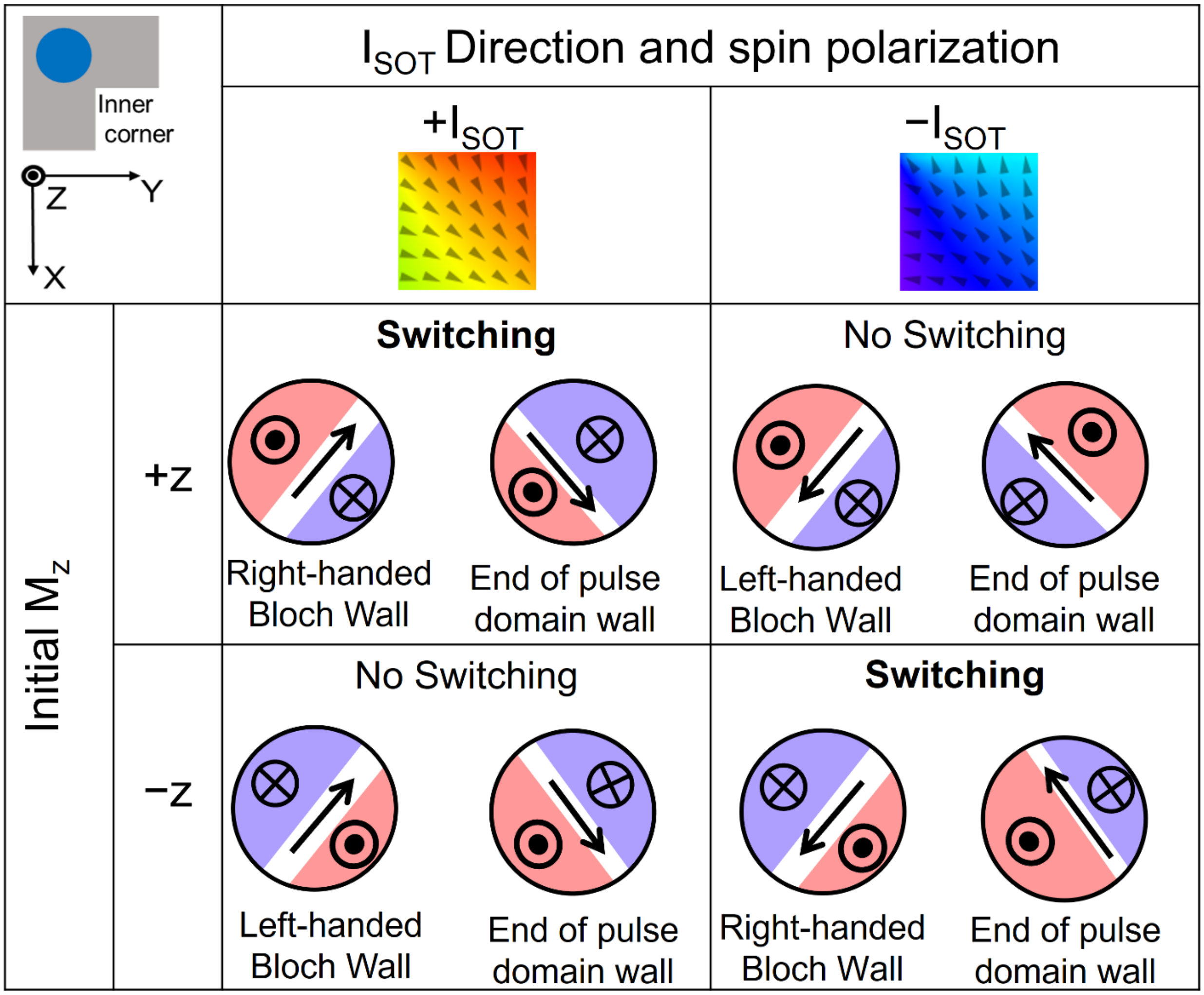


*SI Table 3*. Domain wall propagation during the current pulse for all the combination $I_{SOT}$ and initial $M_z$.

To understand the root cause for time reversal asymmetry in bending structures, we segregate the contribution of both these effects and simulate switching of bending structures either with only spatially varying spin current amplitude below the MTJ (SI Figure 6a and c) or inhomogeneous spin polarization (SI Figure 6b and d). In the case of inhomogeneous spin current amplitude, we observe deterministic nucleation at the inner corner for both directions of current, confirming our claim that the fixed nucleation site is decided by the higher magnitude of spin current at the inner corner. However, the is no domain wall propagation post nucleation and after the pulse is turned off the magnetization relaxes back to its initial state.

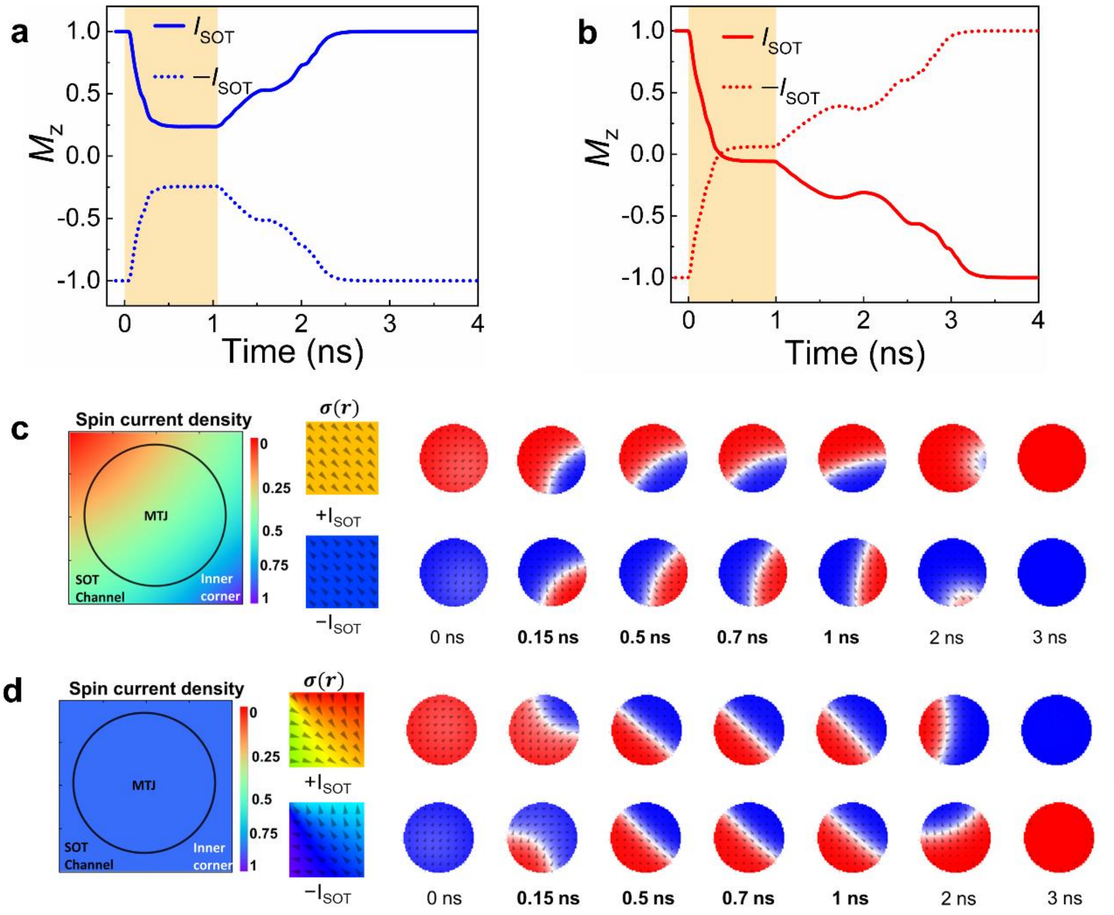


*SI Figure 6. Influence of inhomogeneous spin current and spin polarization on field free switching. (a and b)* Magnetization trace for both directions of current with (a) only spin current gradient *(b) only spatial variation of spin polarization direction. (c and d)* Magnetization Snapshot for direction of current with (c) only spin current gradient *(d)* only spatial variation of spin polarization direction.

In the case of inhomogeneous spin polarization, the nucleation point is determined by SOT physics (DMI sign, direction of SOT field, and current direction[7]). The spatially varying SOT causes the domain wall magnetization to propagate such that it orients along the spin polarization. This leads to a larger portion of FL magnetization to have orientation like the initial nucleated domain thus, helping in differentiation between both the transitions and allowing the system to end in the desired state for both directions of current.

DMI plays a crucial role in domain wall propagation and post-pulse relaxation. To elaborate on the role of DMI on the switching trajectory, we varied the direction and strength of DMI for 0.9 mA (SI Figure 7a) and −0.9 mA (SI Figure 7b) for initial $M_z$ +z direction. For $I_{SOT}$ = 0.9 mA, we observe switching only when the system has a positive DMI. Note that we quantified DMI for the W/CoFeB systems (not shown in the work), and we recorded a small positive DMI of

0.06 mJ/m$^2$. When $I_{SOT}$ = −0.9 mA is applied, we observe an opposite DMI chirality enabling switching compared to $I_{SOT}$ = +0.9 mA. The combination of DMI chirality and SOT induced due to the spatial variation of spin polarization decides the switching polarity thus enabling deterministic switching in bending structures. When the magnitude of positive (negative) DMI is increased for $I_{SOT}$ > 0 ($I_{SOT}$ < 0), we observe a larger region of FL with the same magnetic orientation as the nucleated domain at the end of the current pulse. This further confirms that DMI also plays a key role in the dynamics of bending structure. The switching polarity in bending structures can controlled by the interplay of spin Hall angle sign, the sign of DMI, and the angle of rotation of charge current in the bending structures. Therefore, material selection can further optimize the FFS with bending current.

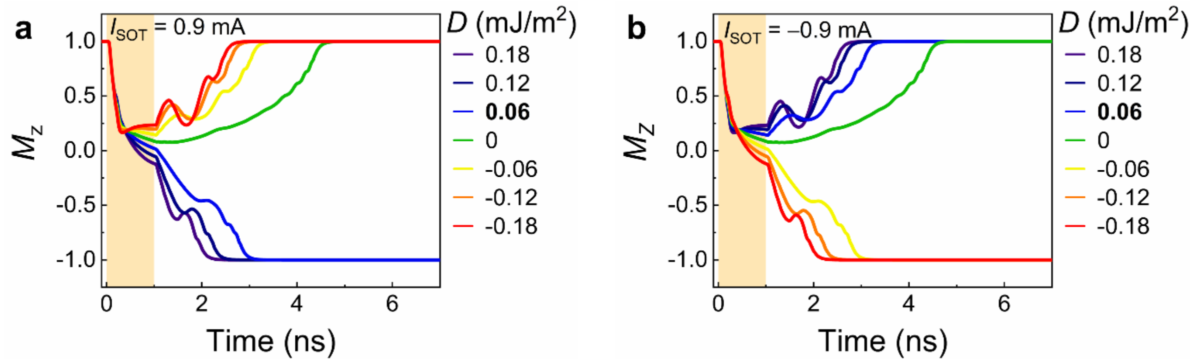


*SI Figure 7. Influence of DMI chirality on field-free switching. (a and b)* Deterministic switching for positive DMI and an increase of the switching speed with the increase of DMI magnitude is observed for $I_{SOT}$ of *(a)* 0.9 mA and *(b)* 1.3 mA.

---

ii. Switching robustness of bending structures

The system constantly switches from AP-P (P-AP) for $I_{SOT} > 0$ ($I_{SOT} < 0$) reliably after the pulse is turned off. To evaluate the robustness of the switching, we varied the pulse rise time and the pulse duration, as well as the size of the MTJ pillar and its position on the SOT channel, and we introduced random thermal fluctuations to the simulations.

Firstly, the rise time for the applied current pulse varies from 0.01 ns to 0.5 ns for a pulse with 1-ns full width at half maximum, showing no impact on the switching outcome (SI Figure 8a). Secondly, the switching trajectory is similar for a broad range of pulse width with fixed $I_{SOT}$ = +0.9 mA (SI Figure 8b) with nucleation of reverse domain immediately after the onset of pulse followed by post-pulse relaxation to the desired state. Thirdly, to account for lithographic error in the placement of the MTJ across the SOT channel, we performed a switching simulation at the same current when varying the location of MTJ up to ± 14 nm along the SOT channel diagonal (as shown in SI Figure 8c). We observe deterministic switching in all the cases indicating that

the creation of inhomogeneous SOT across the FL is more important than the magnitude of SOT. Fourthly, we

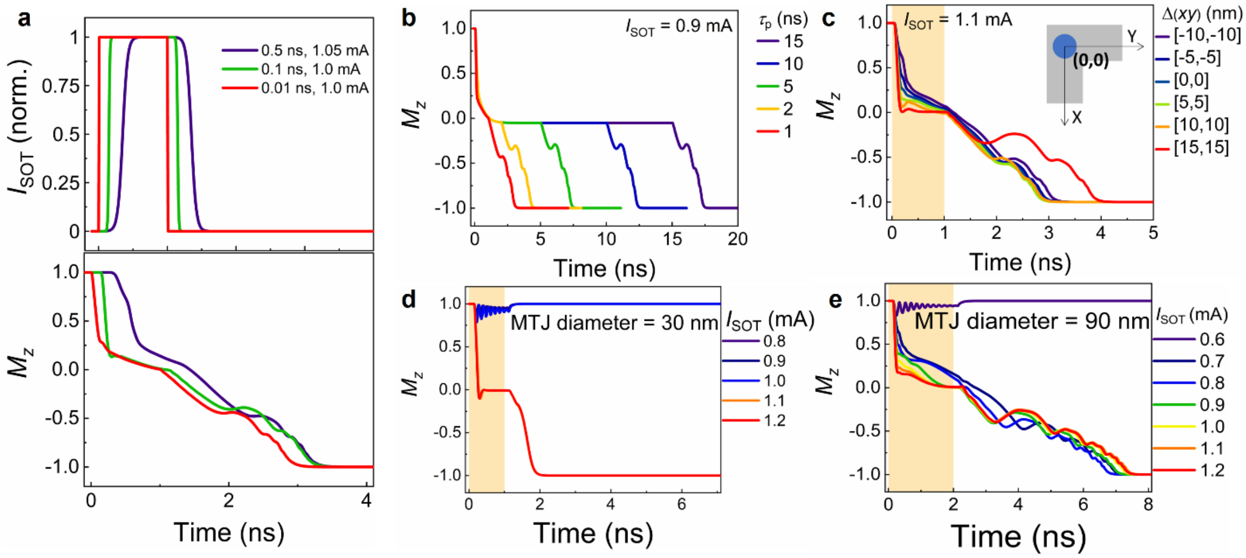


*SI Figure 8. $M_Z$ time trace for bending structures (a)* Switching from the initial AP state for current pulses with different rise times (the top plot shows the normalized current profiles). *(b)* Deterministic switching for various pulse widths for $I_{SOT}$ =0.9 mA. *(c)* Robust switching to P state for $I_{SOT}$ =1.1 mA for different locations of the MTJ on the SOT channel. *(d and e)* Switching for various SOT currents for MTJ of diameter *(d)* 30 nm and *(e)* 90 nm.

observe FFS for a broad range of $I_{SOT}$ for MTJ size of 30 nm (SI Figure 8d) and 90 nm (SI Figure 8e) with a similar trajectory as observed for 60 nm devices. Lastly, we investigate the contribution of thermal fluctuation on the switching of bending structures by introducing the thermal field according to Brown's theory[8] and recording the time trace multiple times. The temperature is introduced in the model as a white noise term $B_{thermal}$ added to the effective field acting on the magnetization in the LLG equation, defined by $\propto \sqrt{\frac{2T\alpha k_B\,\mu_0}{B_{sat}\gamma_{LL}\Delta V\,\Delta t}}$; where $\alpha$ is the damping parameter, $k_B$ is the Boltzmann constant, T the temperature, $B_{sat}$ is the saturation magnetization, $\gamma_{LL}$ is the gyromagnetic ratio , $\Delta V$ is the cell volume, and $\Delta t$ is the time step. The $\vec{\eta}(step)$is evaluated in each simulation step as a Gaussian distribution multiplied by the noise.

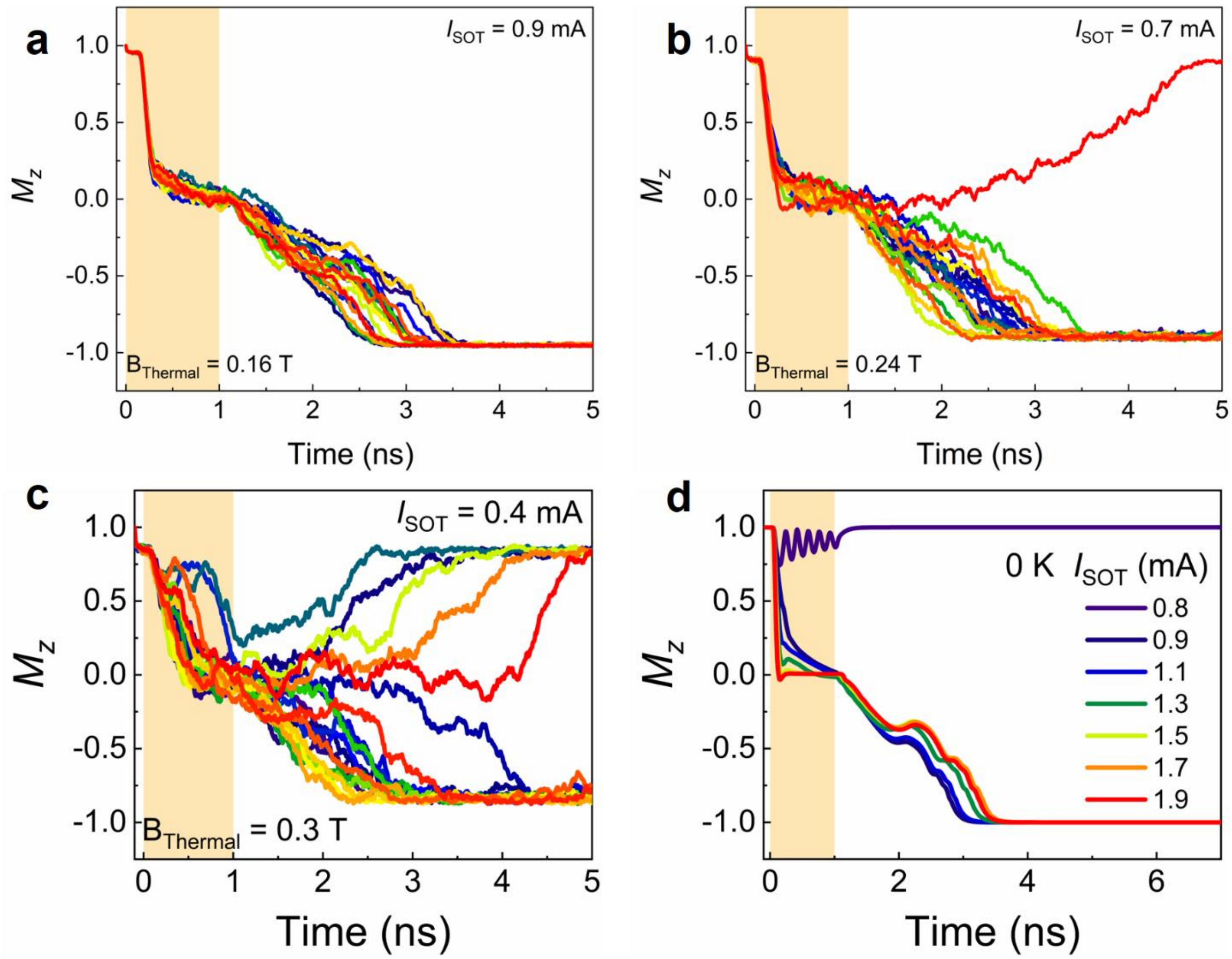


*SI* Figure *9. Impact of temperature on switching of bending structures. (a, b, and c)* 20 $M_Z$ time traces in presence of a stochastic thermal field at (a) 0.16 T and (b) 0.24 T. *(c)* 0.3 T *(c)* Deterministic switching for various $I_{SOT}$ at 0 K.

amplitude term above. The switching trajectory for 20 traces with mean thermal field ($B_{Thermal}$) of 0.16 T (SI Figure 9a), 0.24 T (SI Figure 9b) and 0.3 T (SI figure 9c) are sufficiently close in average to 0 K simulations (SI Figure 9d) to still have well-defined nucleation determined by the spin current gradient, the inhomogeneous spin polarization and DMI contributing for the domain propagation and relaxation. The $B_{Thermal}$ of 0.16 T, 0.24 T and 0.3 T corresponds to approximately 100 K, 200 K and 300 K, respectively. The increase in the random thermal field will reduce the critical switching current. The failed events at higher $B_{Thermal}$ can be attributed to an overestimation of the oscillations due to magnon scattering known to occur in micromagnetic at higher effective temperatures[9,10]. Furthermore, we observed reliable switching experimentally at room temperature, which qualitatively matches the trends observed at 0 K and lower thermal fields.

iii. Change in simulation parameters.

All the simulations for bending structure were performed with a damping parameter of 0.1 and a $B_{FL}/B_{AD}$ ratio of -0.3[2]. To further investigate the role of these parameters, the magnitude is varied for large $I_{SOT}$. Increasing the magnitude of a damping parameter (SI Figure 10a) only increases the post-pulse dynamics indicating no role of damping in the symmetry breaking of the bending structures. The magnitude of the $B_{FL}$ affects the switching threshold (as discussed in the main text). However, it does not impact the nucleation point and has a minor impact on the domain wall propagation during the current pulse. We observed successful switching for all investigated $B_{FL}/B_{AD}$ ratios, as shown in SI Figure 10b.

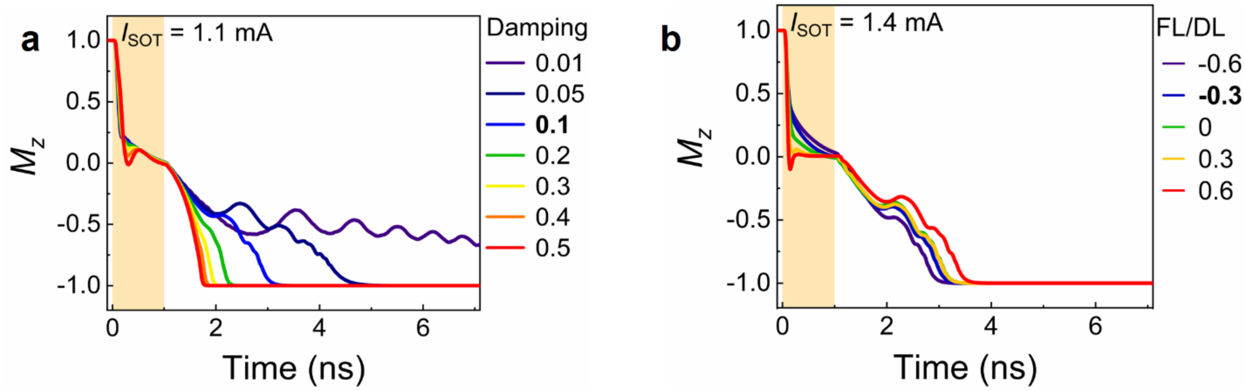


*SI Figure 10. $M_Z$ time trace for non-standard parameters. (a)* Influence of damping on the switching of bending structures. *(b)* The field-like torque's impact on bending structure switching (the standard simulation parameters are highlighted in the legend).

---

In our simulation, we consider the effects of Joule heating and the Oersted field on the FL dynamics. The current flowing in the SOT channel will cause inhomogeneous Joule heating (Joule heating is calculated by measuring change in temperature in the SOT MRAM 3D model in COMSOL), and the resulting inhomogeneous temperature in the SOT channel will impact the magnetic properties of the FL. We account for the variation of saturation magnetization and anisotropy by scaling these parameters as: $M_S(T) = M_{S0}\left(1 - \frac{T}{T_C}\right)^x$ and, $K(T) = K_0\left(\frac{M_S(T)}{M_{S0}}\right)^x$ described in[11] respectively. $M_{S0}$ is the saturation magnetization around 0 K [12], Curie temperature for W/CoFeB/MgO is 750 K, the $Ms$ = 1.1 MA m$^{-1}$ and $Ku$ = 845 kJ m$^{-3}$ at 300 K[12]. As shown in SI Figure 11a, the magnetization switching time traces with and without the spatial variation of magnetic properties of the FL are similar, confirming that the Joule heating does not play a significant role in symmetry breaking for bending structures. Furthermore, inhomogeneous

current induced Oersted field $B_{Oe}$ also does not contribute to the symmetry breaking for bending structures, as there is no change in the trajectory with and without this field (SI Figure 11b).

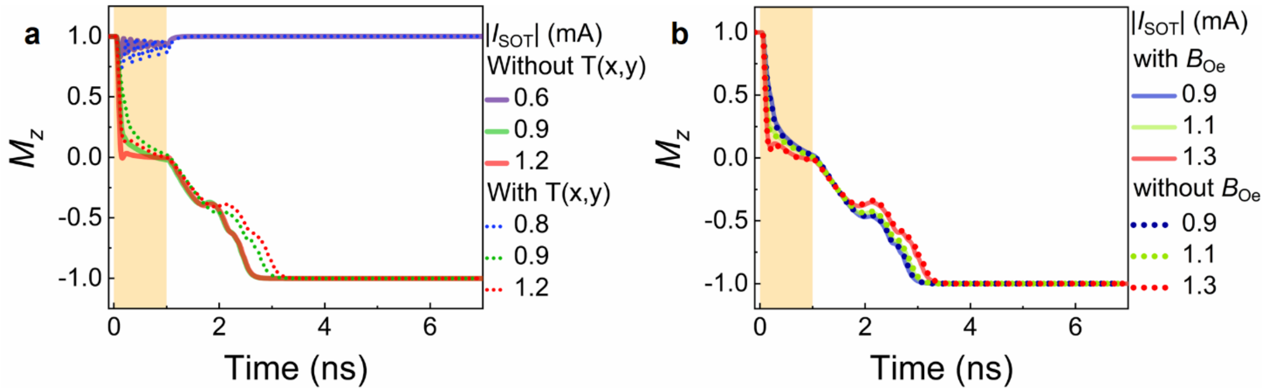


*SI Figure 11. Impact of the secondary effects produced by the large current density on the switching. (a)* $M_z$ trace in the presence and absence of spatial variation of magnetic parameters with T(x,y) induced by the inhomogeneous Joule heating in the SOT channel. *(b)* Switching with and without the contribution of Oersted field generated by the inhomogeneous current distribution in the SOT channel.

# V. Influence of SAF Hard layer

Bending structure switching is sensitive to the stray field generated from the hard layer of the MTJs; for SAF magnetized in +z direction (SAF up), as shown in SI Figure 12a, bending structure switches deterministically in the absence of $B_{ip}$ and $B_z$ (SI Figure 12b). A similar trend is also observed for straight structures when $B_{ip} = -15$ mT is applied for SAF up (SI Figure 12c). However, when the hard layer is reversed (SAF down), as shown in SI Figure 12d, a strong $B_z = 50$ mT is needed to achieve switching in the absence of an in-plane field for bending structures (SI Figure 12e). The range of the z field in which switching occurs is narrow, thus confirming stray field influences the switching in the bending structures. For straight structures with SAF down orientation (SI Figure 12f), we observe that the −z field does not allow bipolar switching even in the presence of $B_{ip} = -15$ mT. The role of the stray field in the switching of bending and straight structures can be explained by the competition/ assistance of the stray field on the $B_{AD}$ experienced by the magnetization of the domain wall during the reversal process (as shown in SI Table 4). The magnetization of the domain wall is influenced by DMI, external magnetic field and direction of current [13]. Considering right-handed DMI and positive current, we see the stray field supports (opposes) expansion of the P state for AP-P transition in the SAF up (SAF down) configuration of the hard layer MTJ. Therefore, the influence of SAF orientation can be reduced in straight structures with an increase in the $B_{ip}$ such that it is higher than the in-plane fringing field generated by the hard layer.

<table>
<tr><td>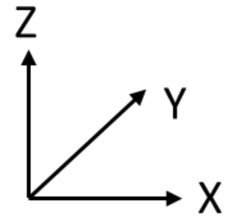</td><td colspan="2">SAF up<br>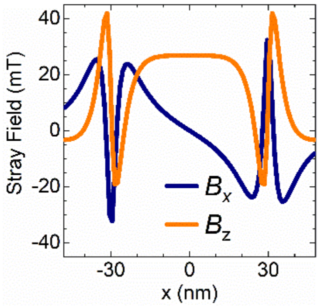</td><td colspan="2">SAF Down<br>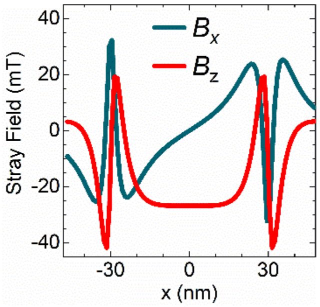</td></tr>
<tr><td>Switching direction</td><td>AP-P<br>(-z to +z)</td><td>P-AP<br>(+z to -z)</td><td>P-AP<br>(-z to +z)</td><td>AP-P<br>(+z to -z)</td></tr>
<tr><td>Current</td><td>+x</td><td>-x</td><td>+x</td><td>-x</td></tr>
<tr><td>Spin polarization (σ)</td><td>-y</td><td>+y</td><td>-y</td><td>+y</td></tr>
<tr><td>Stray field</td><td>+z</td><td>+z</td><td>-z</td><td>-z</td></tr>
<tr><td>Magnetization of the Domain wall ($M_{DW}$)</td><td>+x<br>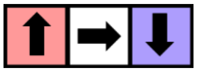</td><td>-x<br>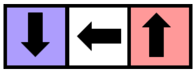</td><td>+x<br>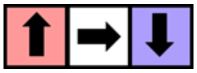</td><td>-x<br>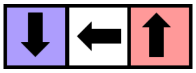</td></tr>
<tr><td>$\mathbf{B}_{AD}$ (along $\boldsymbol{\sigma}$ x $M_{DW}$)</td><td>+z</td><td>+z</td><td>+z</td><td>+z</td></tr>
<tr><td>Stray field and $\mathbf{B}_{AD}$</td><td>Add up</td><td>Add up</td><td>Oppose</td><td>Oppose</td></tr>
</table>

*SI Table 4*. Impact of stray field on the SOT switching.

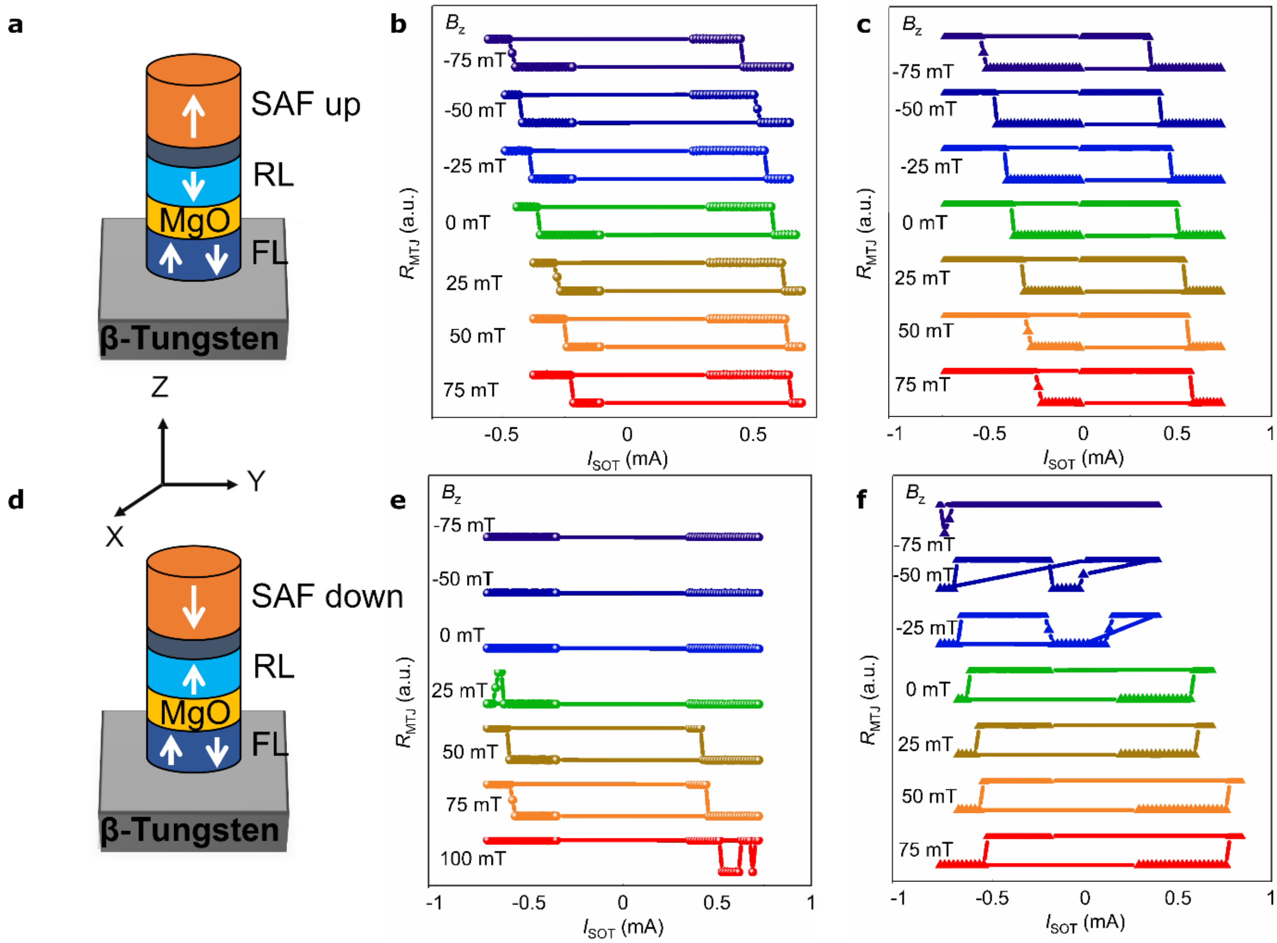


*SI Figure 12. Impact of SAF orientation on switching. (a and d)* Schematic of MTJ with SAF hard layer saturated in *(a)* up (+z) direction and *(d)* down (–z) direction. *(b and c)* $R_{MTJ}$-$I_{SOT}$ loops at the varied magnitude of $B_z$ for SAF up *(b)* bending structures, *(c)* straight structures at $B_{ip}$ = –15 mT. *(e and f)* $R_{MTJ}$-$I_{SOT}$ loops at the varied magnitude of $B_z$ for SAF down *(e)* bending structures, *(f)* straight structures $B_{ip}$ = –15 mT.

# VI. Bending structure design analysis

We perform a design-to-technology co-optimization (DTCO) analysis comparing the bending structures for the 5nm technology node against other embedded memory technologies such as static-RAM (SRAM), STT-MRAM, and SOT-MRAM. In SI table 5, the performance and energies of SRAM high-density (HD) and high-performance (HP) are estimated using our in-house libraries. A 45 nm gate pitch and a 30 nm metal pitch design are used in our technology models to optimize STT-MRAM and SOT-MRAM performances. The power consumptions are estimated using a 128 kbit memory size with 64-bit output[14]. We observe that the bending structures do not compromise on the area compared to a standard SOT-MRAM, as SOT channel dimensioning plays a minor role in determining the area of the SOT-MTJ cell compared to other

factors like the distance between the VIA contacts, the pitch for the various metal lines in the design and the dimension of the transistor. The top and side view of bending and straight structures shown in SI Figure 13 indicate no difference in the area for the bit cell. As seen in the SI Figure 1a, our present design has the MTJ closer to the outer corner of the SOT channel, and the SOT channel is also long. This causes a higher resistance of the SOT channel in our present design, thus needing a higher voltage to control the transistor ($V_{DD}$) and read/write power. To reduce the switching current, we analyzed the impact of the location of the MTJ on the SOT channel by performing a COMSOL simulation with MTJ on two locations on the SOT channel. The comparison between the two locations is made by calculating the current density when the MTJ is closer to the inner and outer corner of the SOT channel (SI Figure 14a). We need to apply a bias of 2.5 V experimentally in the current device, whereas the optimized device needs 1.7 V to achieve a similar current density (SI Figure 14b). This indicates that the switching current decreases by ~ 32% if the MTJ is closer to the inner corner. When the DTCO analysis of this optimized structure is performed, it shows similar properties as straight structures with the added benefit of FFS. The bending structures with the MTJ closer to the inner corner and reduced SOT channel dimension will help in improving the switching properties.

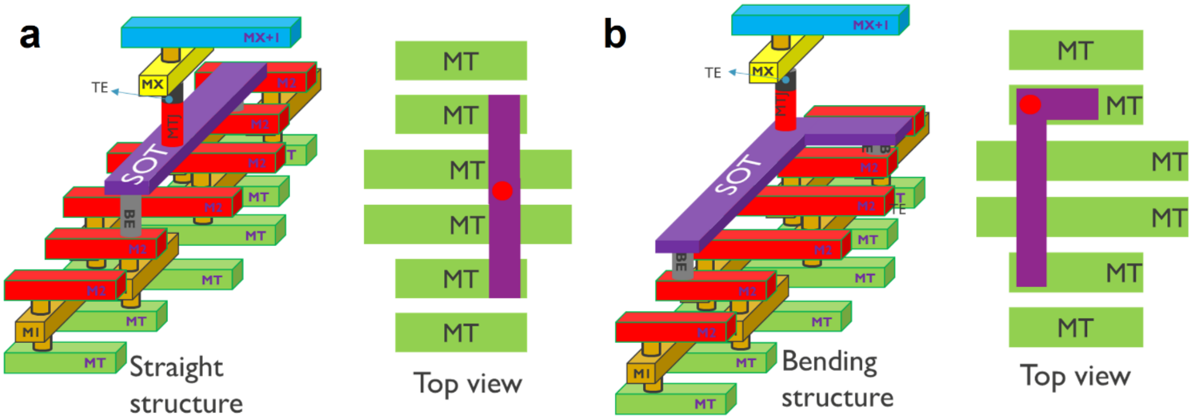


*SI Figure 13.* Design of SOT-MRAM showing all the metal lines with electrodes. *(a)* Straight structures, *(b)* bending structures

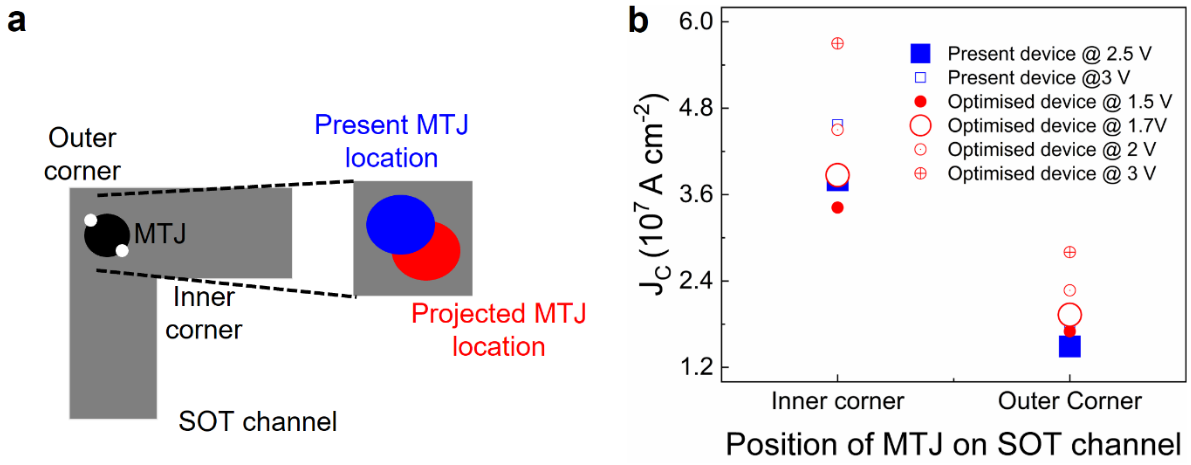


*SI* Figure *14. Impact of the MTJ location in bending structure. (a)* Schematic of SOT channel and MTJ showing the inner corner, outer corner, and location of MTJ on SOT channel. *(b)* Current density on MTJ inner corner and outer corner for different $V_{SOT}$ for present and projected MTJ location.

| Specs | Bit-Cell Types | | | | | |
|---|---|---|---|---|---|---|
| | SRAM | | STT | SOT | Bending structure | |
| | 111 | 122 | | 5T | 5T | Optimized |
| Area ($\mu m^2$) | 0.021 | 0.028 | 0.0084 | 0.0162 | 0.0162 | 0.0162 |
| RD Power per bit ($\mu$W/bit access) | 16 | 30 | 17 | 28 | 32 | 32 |
| WR Power per bit ($\mu$W/bit access) | 21 | 40 | 50 | 73 | 383 | 63 |
| RD latency (ns) | ~1 | ~0.70 | ~5 | ~1 | ~3 | ~3 |
| WR latency (ns) | ~1 | ~0.70 | ~20.00 | ~1.4 | ~5 | ~5 |
| Endurance | ~$10^{16}$ | ~$10^{16}$ | ~$10^{9}$ | ~$10^{14}$ | ~$10^{14}$ | ~$10^{14}$ |
| $V_{DD}$ (V) | 0.7 | 0.7 | 0.7 | 0.7 | 3.5 | 0.7 |

*SI Table 5.* Design-to-technology co-optimization analysis for bending structures.

## REFERENCES

(1) Thomas, L.; Jan, G.; Zhu, J.; Liu, H.; Lee, Y.-J.; Le, S.; Tong, R.-Y.; Pi, K.; Wang, Y.-J.; Shen, D.; He, R.; Haq, J.; Teng, J.; Lam, V.; Huang, K.; Zhong, T.; Torng, T.; Wang, P.-K. Perpendicular Spin Transfer Torque Magnetic Random Access Memories with High Spin

Torque Efficiency and Thermal Stability for Embedded Applications (Invited). *J. Appl. Phys.* **2014**, *115* (17), 172615. https://doi.org/10.1063/1.4870917.
(2) Vudya Sethu, K. K.; Ghosh, S.; Couet, S.; Swerts, J.; Sorée, B.; De Boeck, J.; Kar, G. S.; Garello, K. Optimization of Tungsten β -Phase Window for Spin-Orbit-Torque Magnetic Random-Access Memory. *Phys. Rev. Appl.* **2021**, *16* (6), 064009. https://doi.org/10.1103/PhysRevApplied.16.064009.
(3) Garello, K.; Yasin, F.; Couet, S.; Souriau, L.; Swerts, J.; Rao, S.; Van Beek, S.; Kim, W.; Liu, E.; Kundu, S.; Tsvetanova, D.; Croes, K.; Jossart, N.; Grimaldi, E.; Baumgartner, M.; Crotti, D.; Fumemont, A.; Gambardella, P.; Kar, G. S. SOT-MRAM 300MM Integration for Low Power and Ultrafast Embedded Memories. In *2018 IEEE Symposium on VLSI Circuits*; IEEE: Honolulu, HI, 2018; pp 81–82. https://doi.org/10.1109/VLSIC.2018.8502269.
(4) Raymenants, E.; Bultynck, O.; Wan, D.; Devolder, T.; Garello, K.; Souriau, L.; Thiam, A.; Tsvetanova, D.; Canvel, Y.; Nikonov, D. E.; Young, I. A.; Heyns, M.; Soree, B.; Asselberghs, I.; Radu, I.; Couet, S.; Nguyen, V. D. Nanoscale Domain Wall Devices with Magnetic Tunnel Junction Read and Write. *Nat. Electron.* **2021**, *4* (6), 392–398. https://doi.org/10.1038/s41928-021-00593-x.
(5) Vansteenkiste, A.; Leliaert, J.; Dvornik, M.; Helsen, M.; Garcia-Sanchez, F.; Van Waeyenberge, B. The Design and Verification of MuMax3. *AIP Adv.* **2014**, *4* (10), 107133. https://doi.org/10.1063/1.4899186.
(6) Grimaldi, E.; Krizakova, V.; Sala, G.; Yasin, F.; Couet, S.; Sankar Kar, G.; Garello, K.; Gambardella, P. Single-Shot Dynamics of Spin–Orbit Torque and Spin Transfer Torque Switching in Three-Terminal Magnetic Tunnel Junctions. *Nat. Nanotechnol.* **2020**, *15* (2), 111–117. https://doi.org/10.1038/s41565-019-0607-7.
(7) Baumgartner, M.; Garello, K.; Mendil, J.; Avci, C. O.; Grimaldi, E.; Murer, C.; Feng, J.; Gabureac, M.; Stamm, C.; Acremann, Y.; Finizio, S.; Wintz, S.; Raabe, J.; Gambardella, P. Spatially and Time-Resolved Magnetization Dynamics Driven by Spin–Orbit Torques. *Nat. Nanotechnol.* **2017**, *12* (10), 980–986. https://doi.org/10.1038/nnano.2017.151.
(8) Vansteenkiste, A.; Leliaert, J.; Dvornik, M.; Helsen, M.; Garcia-Sanchez, F.; Van Waeyenberge, B. The Design and Verification of MuMax3. *AIP Adv.* **2014**, *4* (10), 107133. https://doi.org/10.1063/1.4899186.
(9) Barker, J.; Bauer, G. E. W. Semiquantum Thermodynamics of Complex Ferrimagnets. *Phys. Rev. B* **2019**, *100* (14), 140401. https://doi.org/10.1103/PhysRevB.100.140401.
(10) Berkov, D. V. Magnetization Dynamics Including Thermal Fluctuations: Basic Phenomenology, Fast Remagnetization Processes and Transitions Over High-Energy Barriers. In *Handbook of Magnetism and Advanced Magnetic Materials*; Kronmüller, H., Parkin, S., Eds.; John Wiley & Sons, Ltd: Chichester, UK, 2007; p hmm204. https://doi.org/10.1002/9780470022184.hmm204.
(11) Moreno, R.; Evans, R. F. L.; Khmelevskyi, S.; Muñoz, M. C.; Chantrell, R. W.; Chubykalo-Fesenko, O. Temperature-Dependent Exchange Stiffness and Domain Wall Width in Co. *Phys. Rev. B* **2016**, *94* (10), 104433. https://doi.org/10.1103/PhysRevB.94.104433.
(12) Lee, K.-M.; Choi, J. W.; Sok, J.; Min, B.-C. Temperature Dependence of the Interfacial Magnetic Anisotropy in W/CoFeB/MgO. *AIP Adv.* **2017**, *7* (6), 065107. https://doi.org/10.1063/1.4985720.

(13) Emori, S.; Bauer, U.; Ahn, S.-M.; Martinez, E.; Beach, G. S. D. Current-Driven Dynamics of Chiral Ferromagnetic Domain Walls. *Nat. Mater.* **2013**, *12* (7), 611–616. https://doi.org/10.1038/nmat3675.
(14) Wu, Y. C.; Garello, K.; Kim, W.; Gupta, M.; Perumkunnil, M.; Kateel, V.; Couet, S.; Carpenter, R.; Rao, S.; Van Beek, S.; Vudya Sethu, K. K.; Yasin, F.; Crotti, D.; Kar, G. S. Voltage-Gate-Assisted Spin-Orbit-Torque Magnetic Random-Access Memory for High-Density and Low-Power Embedded Applications. *Phys. Rev. Appl.* **2021**, *15* (6), 064015. https://doi.org/10.1103/PhysRevApplied.15.064015.
